# Computed tomography reconstructions of burrow networks for the Opheliid polychaete, *Armandia cirrhosa*

**DOI:** 10.1038/s41597-024-03557-w

**Published:** 2024-06-26

**Authors:** Rebecca M. Howman, Mark N. Mavrogordato, Fernando Alverez-Borges, Martin Solan

**Affiliations:** 1 Ocean and Earth Science, National Oceanography Centre, Southampton, University of Southampton, Waterfront Campus, European Way, Southampton, SO14 3ZH UK; 2 Québec Océan, Takuvik Joint International Laboratory CNRS, Université Laval, Quebec City, QC Canada; 3 University of Southampton, Engineering and the Environment, Highfield, Southampton, SO17 1BJ UK

**Keywords:** Behavioural ecology, Behavioural methods

## Abstract

The morphology and architecture of structures formed by sediment-dwelling invertebrates, such as excavations or burrows, are often assumed to be characteristic of a given species, consistent across a range of environmental conditions, and used to categorise species contributions to ecosystem functioning. However, very few investigations use non-invasive high-resolution techniques capable of determining fine scale variations in burrow form and complexity, or consider whether or not the form of the burrow is context dependent. Here, we provide replicate high-resolution micro-focus computed tomography data for the complete burrow systems of the Opheliid polychaete, *Armandia cirrhosa*, across a range of salinity and habitat conditions. These data provide reference models which can be used by ecologists investigating intraspecific variation in species traits and organism-sediment interactions and, more generally, by those tasked with pattern and shape recognition of objects that are morphologically highly variable and which adjust their architecture with changing circumstance or context.

## Background & Summary

The vertical exploitation of marine sediments by burrowing marine organisms is vital for sediment stability, biogeochemical cycling, and bentho-pelagic coupling^1^. Whereas the way in which sediment-dwelling invertebrates redistribute sediment particles and porewater fluids below the sediment-water interface has received much attention^2^, the role of biogenic features, such as mounds, pits, burrows and tubes, in shaping species contributions to ecosystems has received much less attention. These features add complexity to habitat, but are not necessarily consistent over time^3^ and can vary with biotic and/or environmental context^4–7^, or reflect differences in lifestyle mode^8^ that, in turn, can exert major influences over ecosystem properties^9^. Modifications to a characteristic architecture may also provide the means to monitor the constancy of species contributions to ecosystems under changing conditions or contexts.

Investigations into the effect of environmental variability on sediment-organism interactions have mostly been limited to two-dimensions^10–12^ and methods to infer the three dimensional structure of burrow networks, such as burrow castings^13,14^ and camera insertion^15^, are comparatively invasive^16^. More recently, the use of high-resolution micro-focus computed tomography (μ-CT) has been used to image, in three dimensions, individual organisms^17,18^, their behaviour^19^, and any associated biogenic structures^20^, allowing quantitative examination of burrow characteristics^21^. The majority of these descriptive studies, however, do not consider abiotic or biotic factors that may influence the morphology of biogenic structures, making it difficult to understand the extent of intraspecific variation and generalise the full functional contribution of species.

Here, we provide high-resolution computed tomography reconstructions of the burrow architecture of the opheliid polychaete, *Armandia cirrhosa* across different habitat and salinity conditions. Surficial sediment (<5 cm depth) was collected from three locations in the UK, Portland Harbour (50.570039 N, 2.449059 W), Eight Acre Pond (50.743298 N, 1.536999 W) and Normandy Lagoon (50.744045 N, 1.531321 W), using a hand deployed van Veen grab (0.045 m^2^). Each batch of sediment was sieved (500 μm) in a seawater bath to remove debris and macrofauna, allowed to settle for 48 hours to retain the fine fraction (<63μm) and homogenised prior to being distributed to transparent square acrylic aquaria (internal dimensions, 22 × 22 mm; 150 mm high, wall thickness 1.7 mm; Alternative Plastics Ltd., UK). We used the same van Veen grab to collect individuals of *Armandia cirrhosa* from Portland Harbour (50.570039 N, 2.449059 W; water depth, ~1 m). Grab returns were wet sieved (500 µm) on site and the retained material was transported in aerated temperature controlled water baths (12 ± 1 °C) to the *Biodiversity and Ecosystem Futures Facility*, University of Southampton. A stereomicroscope was used to identify individuals of *A. cirrhosa*, which were transferred to transparent 10 × 10 cm square aquaria (50 ind. aquaria^−1^) and fed on cultured phytoplankton (50:50 mix, *Tetraselmis* sp. and *Phaedactylum* sp.). These aquaria were continuously aerated and 50% water changes were carried out twice a week.

**Fig. 1:**
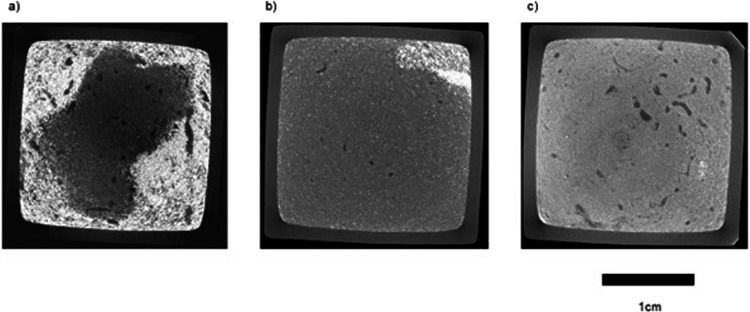
Example coronal plane sections at a horizon ~0.5 cm below the sediment-water interface for *Armandia cirrhosa* burrow networks in sediment obtained from (**a**) Eight Acre pond, (**b**) Normandy Lagoon and (**c**) Portland Harbour along the south coast of England. Burrows appear as darker groupings of grey pixels and particulate luminophore tracers as the lightest grey pixels. Scale bar = 1 cm.

Replicate aquaria, each containing 5 individuals of *A. cirrhosa*, were established for each habitat (7 replicates habitat^−1^; total, n = 21) with a settled sediment depth of 10 cm overlain by 5 cm seawater (~24 ml, salinity 34; 10μm sand filtered, UV sterlised). For salinity, replicate aquaria (5 replicates salinity^−1^; total, n = 35), each containing 1 individual of *A. cirrhosa*, were established for each level of salinity (15, 20, 25, 30, 33, 35, 40) and contained 8 cm depth sediment from Portland harbour overlain by 6 cm seawater. Salinity levels are indicative and reflect the annual range in values observed across the three locations under study. Aquaria were randomly positioned within an insulated water bath maintained at ambient temperature (12 ± 1 °C) with a diurnal photoperiod (18:6 h light-dark regime; BioLumen, Aquabar T-series, Reef Blue light spectra, Tropical Marine Centre, UK). In order to quantify faunal activity, luminophore tracers (dyed particles that fluoresce under ultra violet light, Fig. 1) were added to each aquarium to determine the extent of faunal mediated vertical particle mixing^22^, but these data are not relevant to the 3-dimensional data that are presented here.

Our motivation to provide our μ-CT scans is to encourage investigation into the constancy of functionally important biogenic structures within, and between, species, and across contrasting abiotic and biotic circumstances. Such knowledge will be fundamental in determining and refining more relevant and comprehensive functional effect descriptors that adopt a holistic approach to organism-sediment relations^23,24^, and may be useful in determining the relative performance of species under changing circumstances^25^.

## Methods

**Fig. 2:**
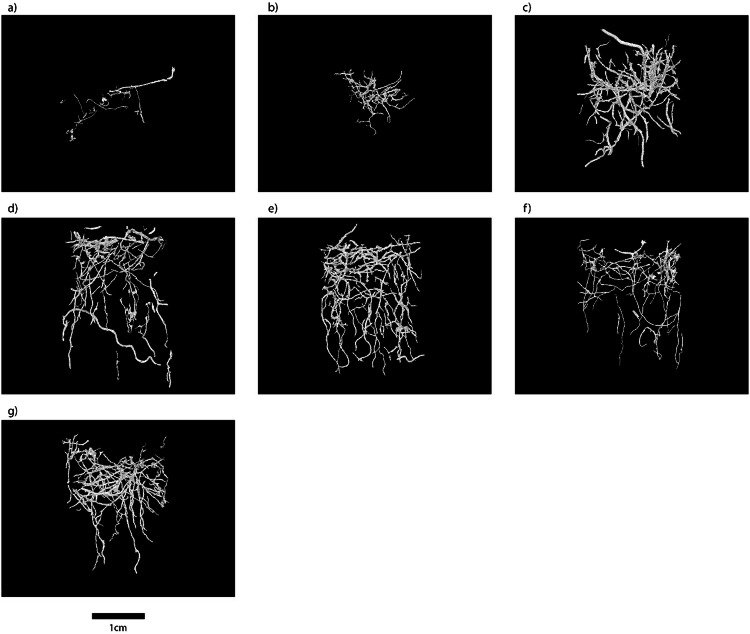
Selected three dimensional reconstructions of burrow networks constructed by *Armandia cirrhosa* obtained from Portland Harbour at a salinity of (**a**) 15, (**b**) 20, (**c**) 25, (**d**) 30, (**e**) 33, (**f**) 35 and (**g**) 40. Scale bar = 1 cm.

We build on the methods and technical validation of our previous work on computed tomography reconstructions of invertebrate burrow systems^26^. Quantification of biogenic structures contained within aquaria (internal dimensions, 22 × 22 mm; 150 mm high, wall thickness 1.7 mm; habitat, n = 3; salinity, n = 35) was achieved using a XT H225 L micro-focus CT system (Nikon Metrology, UK) housed within the μ-VIS imaging centre, University of Southampton. During each acquisition, the aquaria were rotated through 360° whilst collecting 1571 projections, averaging over 16 frames per 88 ms for the habitat samples and 4 frames per 67 ms for the salinity samples. Due to the differences in sample size between the habitat and salinity samples, time per setting was a factor in determining appropriate scan settings. A Tungsten reflection target was used with X-ray conditions set to 160 kV and 189 uA for the habitat samples and 160 kV and 238 µA for the salinity samples, both with no filtration, and an XRD 1621 CN14 HS PerkinElmer flat panel detector was used to collect the images. In the reconstructed images, lighter pixels represent denser material (sediment), and darker pixels represent less dense material (burrow lumen). Each projection was 1000 × 1000 pixels, binned to improve the signal to noise ratio, and to reduce scan times at the expense of spatial resolution. The source to sample distance was 74.87 mm and source to detector distance was 796.48 mm for all samples. This provided a reconstructed voxel size of 37.6 µm. The projection data was reconstructed using CTPro3D (v. XT 2.2 service pack 10, Nikon Metrology, UK) and CTAgent (v. XT 2.2 service pack 10, Nikon Metrology, UK). The reconstructed volumes were converted to 8 bit format using Fiji-ImageJ^26,27^ in order to reduce file sizes and computational loading. Finally, these volume files were analysed in VGStudio (v. 2.1 Volume Graphics GmbH, Germany) and an edge-preserving 3D non-linear digital median filter with a 5 voxel window size was applied to reduce noise in the images. From these data, regions of interest containing the burrows were segmented using a threshold-based seed point growing algorithm to produce 3D burrow volumes^28^ (Fig. 2) and maximum depths (^CT^B_max_, an indication of the extent of vertical influence) of the burrows were calculated.

Global thresholding binarization was carried out on the raw burrow volumes using the interactive thresholding tool in Avizo 3D 2021.2®. Burrow volume (^CT^B_vol_, an indication of ventilatory exchange) was calculated by determining the voxel number withing the burrows using the Global Analysis tool in Avizo 3D 2021.2® to the binarized 3D volumes. The global analysis tool was also used to calculate volume/depth profiles along an x-y plane. Surface area (^CT^B_SA_, an important determinant of microbial-mediated biogeochemical cycling) was calculated by applying the Erosion tool combined with the Subtraction tool in Avizo 3D 2021.2® to the binarized volumes. This eroded a single voxel layer from the surface of the original binarized volumes, and then subtracted the eroded volumes from the binarized volume leaving a single voxel shell. The Global Analysis tool was then used on the single voxel volumes to determine the surface area of the burrows. The Skeletonization tool in Avizo 3D 2021.2® was applied to the binarized volumes to calculate the total cumulative length (^CT^B_L_, an indication of the extent of bioengineering) and mean diameter (^CT^B_⌀_) of the burrow network.

## Data Record

All data records listed in this section are available at Harvard Dataverse^29^. Computed tomography three-dimensional burrow volumes (habitat, n = 3; salinity, n = 35) have been converted into raw.am files, with associated dimensions (volume width, breadth, height) inserted into the file names to enable access by multiple processing programs. Files saved in the raw Avizo format (.am), and all image stacks, can be opened with open source software, such as ImageJ/Fiji using the Bio-Formats plugin. Data tables containing the metrics extracted from the volumes in Avizo are also included as well as movies (.mp4 format) of 3-dimensional burrow structures for all scanned aquaria. Movies of the 3-dimensional burrow reconstructions are provided. ‘*Read Me – Habitat’* and ‘*Read Me – Salinity’* text files outline the details of what each file contains.

## Technical Validation

The system geometry at the μ-VIS X-ray Imaging Centre, University of Southampton, is checked and validated periodically using a 3 ruby sphere reference object that has been measured using optical profilometry (Xyris 4000 CL Surface Profiler, Taicaan technologies Europe). The centroid distances (threshold independent) of these ruby spheres when measured using CT are in agreement with the optical profilometry measurements to within 0.2%. For the presented scans, measurement validation was carried out post-scan by ensuring reference distances were accurately represented in the final images (within 1%).

## Usage Notes

There are no limitations on data use.

## Data Availability

No custom code was used to generate or process the data described in this manuscript.
